# Deep Learning Based on Event-Related EEG Differentiates Children with ADHD from Healthy Controls

**DOI:** 10.3390/jcm8071055

**Published:** 2019-07-19

**Authors:** Amirali Vahid, Annet Bluschke, Veit Roessner, Sebastian Stober, Christian Beste

**Affiliations:** 1 Cognitive Neurophysiology, Department of Child and Adolescent Psychiatry, Faculty of Medicine, TU Dresden, Fetscherstrasse 74, 01309 Dresden, Germany; 2 Artificial Intelligence Lab, Institute for Intelligent Cooperating Systems, Faculty of Computer Science, Otto von Guericke University Magdeburg, Universitätsplatz 2, 39106 Magdeburg, Germany

**Keywords:** ADHD, EEG, deep learning, response selection, attention

## Abstract

Attention Deficit Hyperactivity Disorder (ADHD) is one of the most prevalent neuropsychiatric disorders in childhood and adolescence and its diagnosis is based on clinical interviews, symptom questionnaires, and neuropsychological testing. Much research effort has been undertaken to evaluate the usefulness of neurophysiological (EEG) data to aid this diagnostic process. In the current study, we applied deep learning methods on event-related EEG data to examine whether it is possible to distinguish ADHD patients from healthy controls using purely neurophysiological measures. The same was done to distinguish between ADHD subtypes. The results show that the applied deep learning model (“EEGNet”) was able to distinguish between both ADHD subtypes and healthy controls with an accuracy of up to 83%. However, a significant fraction of individuals could not be classified correctly. It is shown that neurophysiological processes indicating attentional selection associated with superior parietal cortical areas were the most important for that. Using the applied deep learning method, it was not possible to distinguish ADHD subtypes from each other. This is the first study showing that deep learning methods applied to EEG data are able to dissociate between ADHD patients and healthy controls. The results show that the applied method reflects a promising means to support clinical diagnosis in ADHD. However, more work needs to be done to increase the reliability of the taken approach.

## 1. Introduction

Attention Deficit Hyperactivity Disorder (ADHD) is one of the most prevalent neuropsychiatric disorders in childhood and adolescence [1]. Its major symptoms refer to inattention, hyperactivity, and impulsivity [2]. Depending on the strength of these symptoms, different subtypes can be distinguished. The most prominent ones are the inattentive (ADD) and the combined subtype (ADHD-C) [3,4]. Patients with both diagnostic subtypes are characterized by significant attention problems, but only patients with ADHD-C are additionally characterized by impulsivity/hyperactivity. A variety of models try to explain the underlying etiology, with the most widely accepted theories relating to dopaminergic processing and changes in prefrontal cortex functioning [5,6,7]. Currently, the standard diagnostic procedure in ADHD is based on clinical interviews, symptom questionnaires completed by different raters, neuropsychological testing, and the exclusion of other underlying causes that may also lead to the observed symptoms (e.g., other psychiatric conditions, poor vision/hearing, abnormalities in thyroid functioning, or EEG, etc.). In recent years, significant research effort has been undertaken to evaluate the usefulness of neurophysiological (EEG) and functional imaging data to aid this diagnostic process [8,9,10,11,12,13,14,15]. A major problem for this is that there is rarely a one-to-one relationship between neural signals and the phenotypic expression of the disease [16]. However, the advent of machine learning approaches and in particular deep learning using (neuro)physiological data may offer new opportunities for diagnostic purposes [17,18]. Deep learning allows computational models to learn representations of data with multiple levels of abstraction [19] using all information that the dataset has to offer [17,18]. This is a major advantage over more conventional machine learning approaches that only use a small number of features [17]. A small number of studies have so far used deep learning for the classification of EEG data. However, pre-proposed architectures were applied to very specific data sets [20,21,22] and a new deep learning architecture (EEGNet) [23] has been developed in order to decode brain states in Brain–Computer Interfaces.

In the current study, at first taking a three-class problem approach, we ask whether it is possible to use deep learning methods to differentiate between patients with ADHD, those with ADD, and healthy controls. We subsequently further examine those participants who the deep learning architecture is able to classify significantly above the chance level when solving the three-class problem. This was done by turning to a two class-problem, in which we used the same deep learning architecture (EEGNet). For the two-class problem, we asked how well the model is able to distinguish between healthy controls and patients with ADHD or ADD, separately. In addition, we aimed to determine whether it is possible to differentiate between patients diagnosed with different ADHD subtypes. These analyses were based on event-related EEG recording, while ADHD patients and controls performed an interval-timing task. Such tasks examine the precision of executing a motor action after a particular time interval has passed. Since interval timing depends on dopaminergic mechanisms and fronto-striatal networks [24,25,26], it taps into neurobiological processes affected in ADHD very well. Previously, interval timing has reliably been shown to be altered in ADHD [24,27,28,29,30,31,32,33] and seems to be useful to distinguish between ‘real ADHD’ and ‘pseudo-ADHD’ [34]. All these, together with evidence that interval timing processes differ between ADHD subtypes [35], make neurophysiological processes underlying interval timing a suitable starting point to evaluate the capability of deep learning to differentiate between ADHD subtypes and healthy subjects, as well as between the ADHD-C and ADD subtype.

## 2. Experimental Section

### 2.1. Participants

The study was approved by the local ethics committee. Informed written assent/consent was obtained from all participants/their legal guardians in accordance with the Declaration of Helsinki. The ethics committee of the Technical University of Dresden approved the study. The data structure containing all analyzed data can be found at https://osf.io/6594x/. The EEGNet architecture which we used for the data analyses has already been deposited and can be downloaded from https://github.com/vlawhern/arl-eegmodels.

In total, data from *N* = 144 children was included in the analysis (whole sample). *N* = 100 of them were patients with AD(H)D for whom diagnoses had been determined according to standard clinical guidelines by child and adolescent psychiatrists and psychologists using family and school interviews, questionnaires, and IQ and attention testing. Further, possible somatic differential diagnoses were excluded via blood analyses, EEG, audiometry, and vision testing. *N* = 52 participants (10 female, age: 10.9 ± 2.4; IQ: 100 ± 12) fulfilled criteria for ADD according to ICD-10 (F98.8), while the remaining *n* = 48 were diagnosed with the combined subtype (ADHD; ICD-10 F90.0 or F90.1) (12 female, age: 10.6 ± 1.9; IQ: 103 ± 13). The patients revealed no other severe or acute psychiatric co-morbidities (e.g., autism, tics, depressive episode, etc.). The remaining *N* = 44 participants were healthy control children (15 female, age: 11.3 ± 2.2; IQ: 103 ± 12). Using the AD(H)D Symptom Checklist [36], all parents rated their children on a scale of 0 (no problems) to 3 (severe problems) in regards to AD(H)D core symptoms (inattention: controls: 0.5 ± 0.4; ADD: 2.1± 0.43; ADHD: 2.2 ± 0.47; hyperactivity: controls: 0.14 ± 0.3; ADD: 0.7 ± 0.5; ADHD: 1.8 ± 0.6; impulsivity: Controls: 0.4 ± 0.5; ADD: 1.2 ± 0.64; ADHD: 2.4 ± 0.48). Healthy controls had significantly lower scores than the two patient groups on all three subscales (all *F* > 134.8, all *p* < 0.001). Patients with ADD and ADHD did not differ significantly in regards to the degree of inattention (*p* = 0.06). As expected, and based on the different disorder characteristics, hyperactivity (*p* < 0.001) and impulsivity (*p* < 0.001) were significantly more pronounced in patients with ADHD than in those with ADD. Groups did not differ in age, IQ, or gender distribution (all *F* < 1.5, all *p* > 0.3).

After the initial analysis with the whole sample had been performed, we then focused further analyses on those participants for whom the deep learning architecture was able to yield classification accuracy significantly above the chance level. This well-classified sample contained *N* = 18 patients with ADHD, *N* = 15 patients with ADD, and *N* = 25 healthy controls. This sub-sample did not differ from the whole sample in regards to age (*t*(212) = −0.12; *p* = 0.9), IQ (*t*(212) = −0.29; *p* = 0.77) and inattention (*t*(212) = 1.18; *p* = 0.24), hyperactivity (*t*(212) = −0.40; *p* = 0.69), or impulsivity (*t*(212) = 0.71; *p* = 0.48). This was also the case when these comparisons between the whole and the well-classified sample were performed separately for the healthy controls, the patients with ADD, and those with ADHD.

### 2.2. Task

During the task, participants were required to estimate a time of 1200 ms following visual stimulus (white square on black background) [35,37,38]. They were asked to press a button whenever they thought that this time had elapsed. Responses given between 1000 and 1400 ms were regarded as in-time, correct responses. Visual feedback was provided to inform the participants about the correctness of their response. A green “smiley” was shown for correct responses, a red “frowney” together with the statement “too early”/“too late” was presented when responses were given outside of the required time window. The experiment consisted of 300 trials that were equally divided into three blocks. The inter-trial interval was randomized between 800 and 2200 ms. Between the blocks, participants were asked to take a break for one minute.

### 2.3. EEG Recording and Analysis

EEG was recorded from 60 Ag/AgCl electrodes using a BrainAmp amplifier (Brain Products Inc, Munich, Germany). For further analysis, electrodes P9, P10, P11, and P12 were removed from the data set due to their high susceptibility to high electrode impedances and thus unreliable EEG recordings. Thus, 56 electrodes were used for further analysis in deep learning. The sampling rate was 500 Hz and electrode impedances were lower than 5 kΩ. The reference electrode was positioned at Fpz, the ground electrode at coordinates θ = 58, ϕ = 78. Before deep learning was conducted, the standard EEG data pre-processing was run. This included band-pass filtering between 0.5 and 20 Hz (slope of 48 dB/oct). Then, technical artifacts were removed through a raw data inspection. Pulse artifacts and horizontal and vertical eye movements were removed using independent component analysis (ICA, infomax algorithm). This was followed by a segmentation procedure in which the data was stimulus-locked and segmented from −200 ms before to 3000 ms after target onset. The interval from −200–0 ms before the stimulus was used for baseline correction. Afterward, an automated artifact rejection procedure was applied to exclude any remaining trials containing artifacts (amplitude criterion: 200 µV/−200 µV; maximal value difference: 200 μV in a 200 ms interval; low activity: below 0.5 μV in a 100 ms period). A current source density (CSD) transformation was used to allow a reference-free evaluation of the EEG data [39]. These single-trial segmented data were then used for the deep learning approach. For the control group, *N* = 10,129 trials were included, for the ADD group, *N* = 13,031 trials were included, and for the ADHD group, *N* = 10,742 trials were included.

Since low resolution brain electromagnetic tomography (sLORETA) provides a single linear solution for the inverse problem without localization bias [40,41,42], and has been validated by combined fMRI/EEG and TMS/EEG studies [42,43], this method was used for source estimations. For the current study, sLORETA was used to estimate the source of electrical activity, which the deep learning model turned out to be the most predictive for group membership. sLORETA requires standard electrode coordinates according to the 10/10 or 10/20 system as input. sLORETA uses a three-shell spherical head model and the covariance matrix was calculated using the single subject’s baseline. For sLORETA, the intra-cerebral volume is partitioned into 6239 voxels using a spatial resolution of 5 mm and the standardized current density is calculated for every voxel, using an MNI152 head model template. To calculate the statistics on the sLORETA sources, we utilized voxel-wise randomization tests with 2500 permutations and statistical nonparametric mapping procedures (SnPM). Locations of voxels that were significantly different (*p* < 0.01) are shown in the MNI-brain www.unizh.ch/keyinst/NewLORETA/sLORETA/sLORETA.htm. Activations shown in the brain represent critical *t*-values corrected for multiple comparisons.

### 2.4. Deep Learning

As mentioned, we used EEGNet as a deep learning architecture in the current study. The EEGNet performance was previously examined in regards to components such as the P300, visual-evoked potentials, error-related negativity responses (ERN), movement-related cortical potentials (MRCP), and sensory motor rhythms (SMR) [23]. Details of the EEGNet architecture, including the parameters used in this study, can be found in Table 1.

EEGNet consists of two main blocks and the input has a shape (C, T) where C and T represent the number of channels and time points in EEG data, respectively. In the first block, F1 temporal feature maps were produced by applying convolutional filters with a width of 64 samples. After that, for each temporal feature map, D spatial filters spanning all EEG channels were learned by applying depths-wise convolution. Unlike ordinary convolution, which is fully connected into all previous feature maps, depth-wise convolution is connected just in one previous feature map and D is a parameter that controls the number of spatial filters the model must learn for each temporal filter. Therefore, for each temporal feature map, D spatial filters were employed. After applying temporal and spatial filters, batch normalization followed by exponential linear unit (ELU) nonlinearity as an activation function, average pooling over four times steps with a stride of four and dropout were applied. This resulted in outputs with the shape of (F1*D, 1, T//4). In the second block, a separable convolution consisting of depth-wise temporal filters of width 16 followed by point-wise convolution was used. Separable convolution is a very helpful technique since it has fewer parameters than ordinary convolution. Therefore, it is possible to train a model that is less prone to over-fitting. Again, batch normalization followed by ELU activation function, average pooling over eight times steps and dropout were used. Finally, the classification part is done using a dense layer with a softmax activation function.

To apply EEGNet, we first created two-dimensional arrays from every single trial in our data set in which all channels are represented in rows and the time points are represented in columns. Moreover, the batch size was set to 32 and we used the dropout technique with a dropout rate of 0.25. The number of temporal and spatial filters (F1, D) were set to (4, 2), as suggested in Reference [23]. For training, we used the ADAM optimization method [44] and the “leave one out subject” (LOOS)-approach for evaluating the classification performance. In this approach, one subject is selected for testing, while the remaining subjects are used for training the classifier. Moreover, for each group, four subjects from the training subjects were randomly selected as validation subjects. Please note that validation subjects were only used for model selection (i.e., picking the models with lowest cross entropy) and all classification results reported in this paper are based on the test set. The process of randomly selecting, testing, and training subjects was repeated until each subject was used as a test once. We trained for 500 epochs and saved the model with the lowest cross entropy loss in the validation set. After that, the test data was used to evaluate classification performance. To measure classification performance, we calculated the respective mean and standard deviations of classification accuracy among all subjects based on LOOS. It is important to mention that when we want to evaluate classification performance based on the LOOS method, the amount of data in the test set is equal to the number of trials that a subject performed. Therefore, this method is different from “leave one out” (LOO), which just considers one data in the test time. As a result, problems that have been discussed in Reference [45], such as maximizing variance of the test set or overfitting, are less of an issue in LOOS. Finally, the K-fold validation method provides a better estimation of the model’s performance, since it reduces variance in the test set. However, since we want to predict model performance at the subject level, it is necessary to partition the data based on subjects. Using the k-fold method, which partitions data based on a fixed rate, this is not possible. LOOS is comparable to the k-fold method with the difference that we partitioned data based on subjects instead of partitioning it based on a fixed rate.

We applied a class-weight to the loss function since our data is imbalanced (unequal number of trials for each class). The class-weight applied is the inverse of the proportion in the training data, with the majority class set to one. To extract what the model learned best, and which input features (time points/channels) have the highest impact on the classification decision, we employed a “saliency map” approach [41]. In such visualization methods, the aim is to identify the features in each individual data that contribute most to the classification process. In other words, the aim of saliency maps is to determine what input features cause a classifier to make a decision. It is calculated by taking the gradient of the classification score (i.e., before softmax-activation function) with respect to the input data. This provides information about how the output category value (classification score) changes with respect to a small change in input data. By using a saliency map for each input data, we have a visualization map that equals the input feature dimension (i.e., number of channels and time points). In order to provide better visualization of the results (i.e., performance of the applied method), all saliency maps of every single trial belonging to a specific group were averaged and shown in the results section. We also used a normalization method in which the minimum and maximum of the averaged relevance scores were set to zero and one, respectively. Using this approach, values close to one indicate that the specific feature at the specific time points contributes most to classification accuracy

Importantly, to test classification accuracy statistically, we calculated a threshold indicating classification accuracies significantly above the chance level by assuming that classification error obeys a binomial cumulative distribution. As suggested in Reference [46], we used the MATLAB function “binoinv” to compute the statistically significant threshold $std\left(\alpha \right)=binoinv\left(1-\alpha ,n,\frac{1}{c}\right)*\frac{100}{n}$, where *α* is the significance level, *n* is the number of predictions (i.e., number of data in test set) and *c* is the number of classes. This function provides a threshold, which means that a classification accuracy higher than this threshold is considered as significantly above the chance level. Permutation tests are also popular for testing classification accuracy statistically and work based on estimating the error distribution empirically. In permutation tests, we randomly assign a label to each data and train the model to compute classification accuracy. In order to estimate error distribution, we should perform this process for several times (i.e., 1000 times). However, training a deep learning model with lots of parameters such as EEG Net for 1000 times is impractical and very time-consuming. Thus, in this research, we used the binomial cumulative distribution approach to estimate error distribution, which is not time consuming since we do not need to train the model. As mentioned in Reference [46], this method is particularly well suited for large sample size (i.e., N~100). A comparative study showed no major differences between permutation testings and the binomial method we used [46]. Since the number of samples in the test set is equal to the number of trials that a subject performed (235 ± 50 in the current study; i.e., greater than 100), both permutation and binomial, result in similar significant chance levels.

## 3. Results and Discussion

### 3.1. Behavioural Analysis

Behaviorally, both, patients with ADD (47 ± 17%; *p* ≤ 0.001) and patients with ADHD (47 ± 15; *p* ≤ 0.001) showed significantly fewer correctly timed responses than healthy controls (64 ± 11%; F(2,137) = 20.2; *p* ≤ 0.001). This is in line with previous findings (Bluschke et al., 2018b). The whole and the well-classified sample did not differ in regards to behavioral performance (all *p* > 0.20). This was also the case when the healthy controls, the patients with ADD, and those with ADHD were considered separately.

### 3.2. Deep Learning Results

**Figure 1:**
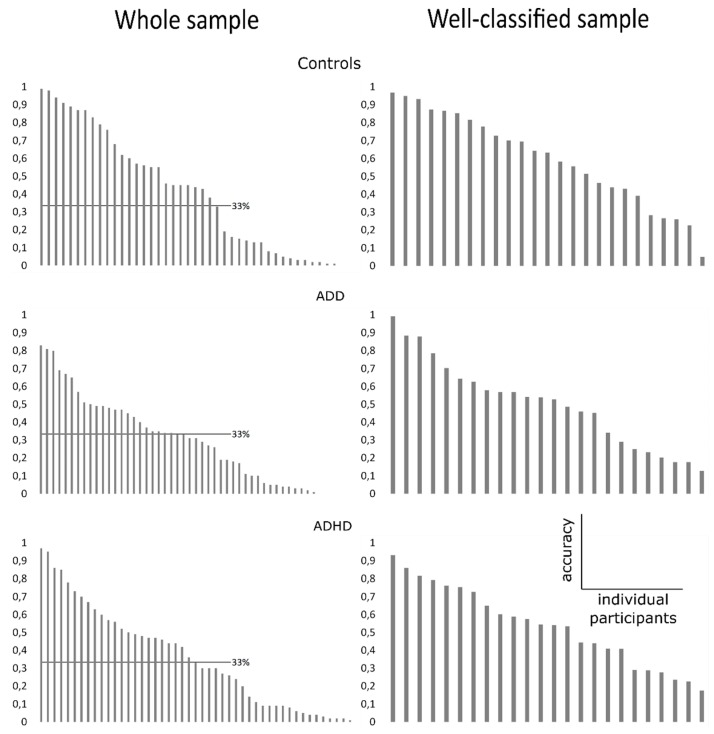
Plots showing distribution of classification accuracy in the whole sample (**A**) and in the well-classified sample (**B**) for all three groups. Y-axis indicates classification accuracy, x-axis indicates the individual participants ranked by classification accuracy (from highest to lowest). In (**A**), the single-trial EEG data recorded during the time estimation was used to classify each individual participant as belonging to the healthy control, the Attention Deficit Disorder (ADD), or the Attention Deficit Hyperactivity Disorder (ADHD) group. To account for potential artifacts and outliers, any participants with a classification accuracy below chance level (33%, as indicated by vertical line) were not included in subsequent analyses. In (**B**), the same analysis of classification accuracy was run a second time. Here, only the single-trial EEG data of participants belonging to the well-classified sample were used. Importantly, the plots show the distribution of classification accuracy.

We used LOOS for evaluating the classification performance of EEGNet in distinguishing patients with ADHD/ADD from healthy controls using single-trial EEG data (three-class problem). The data showing the accuracy to classify the individual participant’s ‘identity’ correctly (i.e., as belonging to the control, the ADD or the ADHD group) on the basis of the single-trial EEG data from these individual subjects are shown in Figure 1A. The mean and standard deviation of accuracy are 39% and 30%, respectively.

As can be seen in Figure 1, the classification accuracy using single-trial EEG data of the whole sample varied considerably between subjects and ranged between 1% and 99% classification accuracy. The distribution of classification accuracy was not different between the three tested groups (Kruskal–Wallis test: *H* = 0.78; *p* > 0.676). This shows that the model reveals no accuracy differences between groups to classify the subjects on the basis of the single-trial EEG data. However, only in a subset of subjects, the applied model was able to perform classification above the chance level. For each subject, we computed a threshold (i.e., significantly above chance level) based on the binomial distribution. If the classification accuracy was higher than this threshold, this subject was included in further analyses. An above significant chance level classification was possible in *N* = 25 subjects in the control group, *N* = 15 subjects in the ADD group, and *N* = 17 subjects in the ADHD group. For all of these groups, classification accuracy was significantly above chance level (all *t* > 3.99; *p* < 0.001). The degree of classification accuracy for subjects classified above chance did not differ between the groups, as indicated by the non-significant Kruskal–Wallis test (*H* = 2.55; *p* = 0.231). Thus, the data show that the neurophysiological dynamics revealed by the subjects can only be used for an above-chance classification in a subset of participants and patients.

### 3.3. Classification Accuracy

However, when evaluating whether the model is well able to classify between groups of subjects in those individuals where the model was already able to reliably use the single-trial EEG data to identify the individual’s group membership as significantly above chance level (see above), the following picture emerged: The EEGNet architecture revealed a three class problem classification accuracy of 69 ± 28% for distinguishing patients with ADHD or ADD from healthy controls (see Figure 1 B) (chance level at approximate 33%, see above).

**Figure 2:**
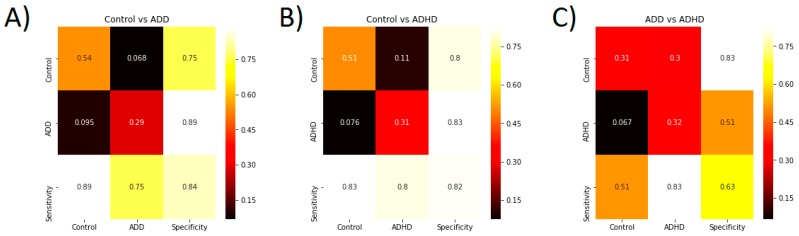
(**A**) Confusion matrix showing the model performance to classify controls vs. ADD patients. (**B**) Confusion matrix showing the model performance to classify controls vs. ADHD patients. (**C**) Confusion matrix showing the model performance to classify ADD vs. ADHD patients. For each plot, the sensitivity and specificity are also provided.

Concerning the two class problem, a classification accuracy significantly above chance level among subjects for Control vs. ADD, Control vs. ADHD, and ADD vs. ADHD lies at 55.5 ± 0.95% 55.5 ± 0.98% and 55 ± 0.6%, respectively. The two class problem classification accuracy for distinguishing patients with ADD or ADHD from healthy control in two class problems is 83 ± 23% and 80 ± 21%, respectively. Confusion matrices for these two analyses are shown in Figure 2.

In both cases, the actual classification accuracy range does not have any overlap with the significant chance level range. Thus, model performance is statistically above chance level. Therefore, specific aspects of single-trial EEG data recorded during a time estimation task can distinguish between healthy controls and patients with a very high accuracy.

In contrast, the two-class problem classification accuracy (67 ± 29%) was very close to chance level (55%) when aiming to distinguish between patients with ADD and those with ADHD. Thus, when the model was aiming to distinguish between patients with ADD and ADHD, there was a large overlap between the actual classification accuracy and the significant chance level. Therefore, single-trial EEG data recorded during a time estimation task cannot reliably distinguish between patients with ADD and ADHD.

### 3.4. Most Important Neurophysiological Features

**Figure 3:**
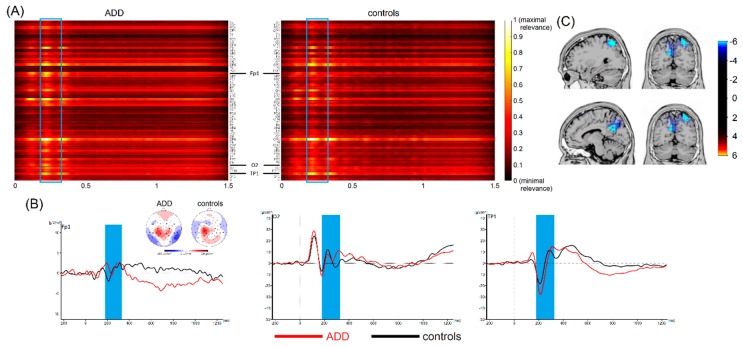
(**A**) Visualization maps showing the relevance of all time points and electrodes for classification between patients with ADD and healthy controls. Values close to 1 indicate that the specific feature at the specific time points contributes most to classification accuracy. The blue frame highlights the most important time range. Based on visual inspection, electrodes Fp1, O2, and TP1 carried the highest relevance. (**B**) Corresponding waveforms (event-related potentials) at the most relevant electrodes are shown with the relevant time range according to the applied machine learning algorithm highlighted in blue. Time point zero denotes stimulus onset in the interval timing task. The black line denotes healthy controls, the red line denotes patients with ADD. Topographic maps show cortical activity in the highlighted time ranges. Blue values denote negativities, red values show positive activations. (**C**) Results of sLORETA source localization show activation in (superior) parietal areas to be different between healthy controls and patients with ADD in the relevant time range.

**Figure 4:**
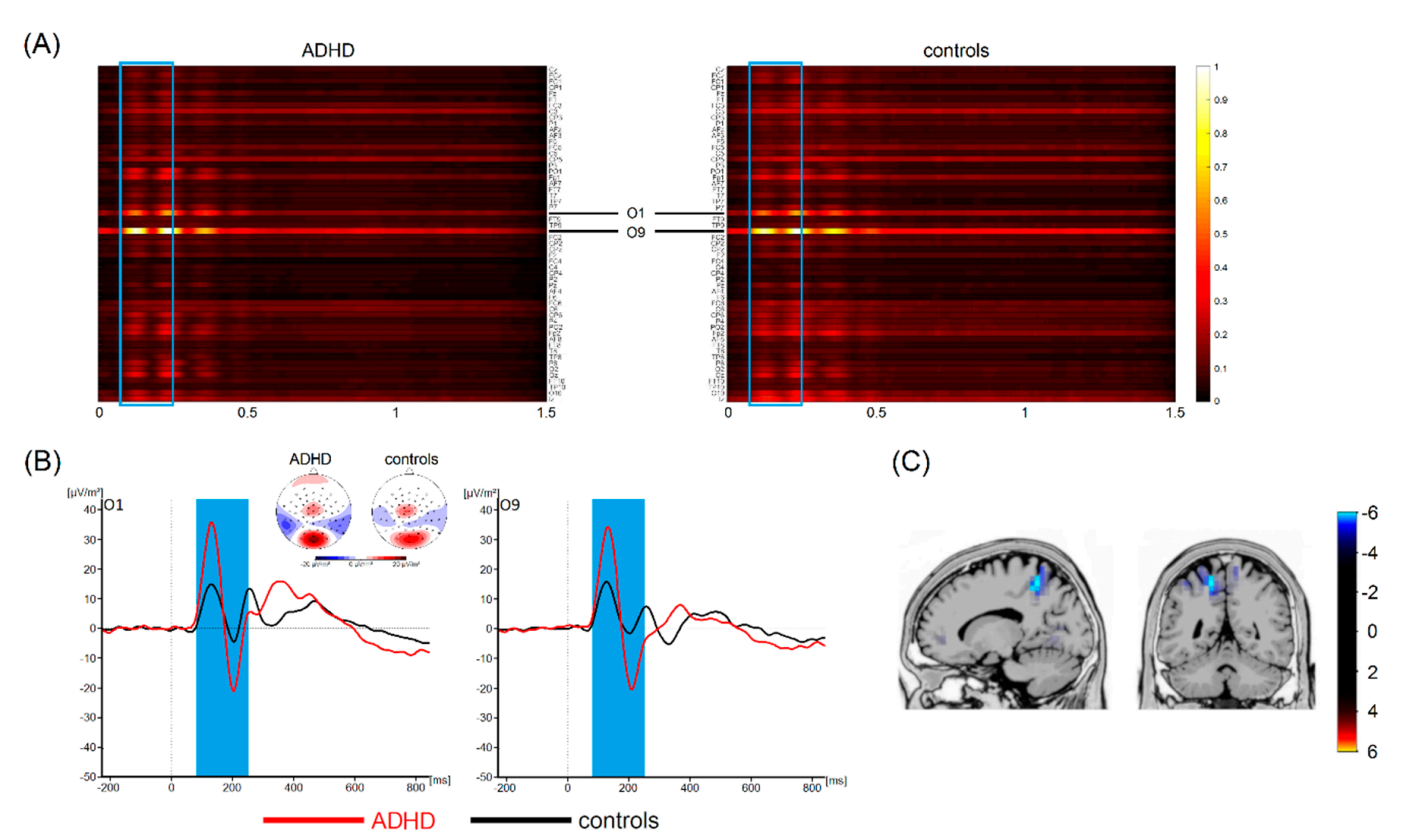
(**A**) Visualization maps showing the relevance of all time points and electrodes for classification between patients with ADHD and healthy controls. Values close to 1 indicate that the specific feature at the specific time points contributes most to the classification accuracy. The blue frame highlights the most important time range. Based on visual inspection, electrodes O1 and O9 carried the highest relevance. (**B**) Corresponding waveforms (event-related potentials) at the most relevant electrodes are shown with the relevant time range according to the applied machine learning algorithm highlighted in blue. Time point zero denotes stimulus onset in the interval timing task. The black line denotes healthy controls, the red line denotes patients with ADD. Topographic maps show cortical activity in the highlighted time ranges. Blue values denote negativities, red values show positive activations. (**C**) Results of sLORETA source localization show for activation in superior parietal areas to be different between healthy controls and patients with ADHD in the relevant time range.

Thus, single-trial EEG data cannot provide sufficient information for discriminating between these two ADHD subtypes. This is the first study that used a deep learning architecture (i.e., EEGNet) [23] to differentiate between patients with different ADHD subtypes and healthy subjects. In contrast to other, more conventional machine learning approaches, deep learning allows computational models to learn representations of data with multiple levels of abstraction [19] using all the information a dataset has to offer [17,18]. As mentioned, in a subset of cases, our analyses revealed very high classification accuracies (around 80%) between healthy controls and patients with ADHD/ADD. However, this was only the case if the classification performance of EEGNet in distinguishing patients with ADHD/ADD from healthy controls using single-trial EEG data was significantly above chance level. In both cases, the deep learning architecture revealed that time ranges between 100 and 200 ms contributed most to the distinction between ADHD/ADD patients and healthy controls (refer to Figure 3 and Figure 4).

These time windows correspond to the time window in classic event-related potential (ERP) where components like the P1, N1, and P2 occur (refer Figure 3 and Figure 4). The source localization revealed that parietal areas (i.e., BA7) and parieto-occipital areas (i.e., the precuneus, BA 19) are associated with these areas (refer Figure 3 and Figure 4). However, especially superior parietal areas were consistently found in each of the examined groups (i.e., ADHD and ADD). It is well-known that the P1 and N1 ERP-components reflect bottom-up perceptual gating and attentional selection processes [47,48,49,50] and that these functions have been shown to be compromised in ADHD [51]. As such, the deep learning architecture used to distinguish ADHD/ADD patients from healthy controls identifies neurophysiological processes associated with attentional processes and superior parietal structures to be of highest relevance. Several lines of imaging research show that superior parietal structures play an important role in ADHD/ADD [52,53] and suggest that there is a good correspondence between anatomical circuitry mediating cognitive functions and patterns of structural changes [52]. Especially superior parietal cortices have been suggested to be important for the representation of task-related signals and for the modulation of incoming information by attention [54]. Based on that, it seems that early sensory and attentive processes are the most important to distinguish between different ADHD subtypes and healthy controls. Interestingly, it has been shown that ERPs in the time windows identified by the applied deep learning approach show a good test–retest reliability in ADHD [55]. It is, however, important to note that previous work did not always reveal differences between ADHD patients and controls when focusing on P1 and N1 ERP-components [56,57,58,59,60]. While this may seem to be at odds with the current findings revealing a high classification accuracy, it is important to consider that the deep learning approach revealed such a high accuracy when considering the entire dataset. While the features in the time interval between 200 and 250 ms strongly contribute to classification accuracy, these alone are not sufficient to achieve high classification accuracy. Relevant time ranges thus also extend to mechanisms of response preparation and timing processes per se, which directly follow the attentional processes. This fits well with findings showing that interval timing processes are particularly altered in ADHD [24,27,28,29,30,31,32,33] and seem useful to distinguish between ‘real ADHD’ and ‘pseudo-ADHD’ [34].

### 3.5. General Discussion

Taking all this together, the current findings suggest that neurophysiological correlates with attentional selection and interval timing in combination with deep learning methods and may prove useful to support a currently applied diagnostic procedure to distinguish ADHD from healthy subjects. Currently, hardly any focus is directed towards these aspects of perception/cognition during the diagnostic processes and the associated clinical assessment methods. Our results suggest that the basic sensory perception processes may, in particular, play a bigger role in this regard than is currently assumed. Thus, it may be important to emphasize these processes during the diagnostic procedure and especially within the associated neuropsychological assessments. It is, however, important to note that the current data cannot answer the question of as to whether this approach is sufficiently specific for diagnostic decisions. This is a limitation of the study. Future studies may also compare different deep learning architectures to evaluate whether there are differences in the sensitivity between algorithms in supporting diagnostic decisions. Further, deep learning algorithms might, in the future, also be used when aiming to distinguish between different psychiatric disorders that are characterized by overlapping symptoms. Deep learning approaches might thus also have the potential to support diagnostic decisions in the clinical setting. However, even though classification accuracy of patients with AD(H)D versus healthy controls reached a satisfactory magnitude in the current study, caution is clearly warranted when discussing the potential applicability of this approach in the clinical setting. Following further methodological refinement, it may be feasible to apply such machine learning-based approaches as one element among the other irreplaceable diagnostic procedures in the future. This may help to reduce variance in the diagnostic process, limit the influence of possible biases, and result in more consistent, valid, and reliable AD(H)D diagnoses [61].

Relating to that, the deep learning algorithm applied in the current study was not able to differentiate between different ADHD subtypes, i.e., classification was at chance level (i.e., 67 ± 29 %), even within the group of subjects in which a reliable classification of controls vs. ADD/ADHD was possible. While this may suggest that EEGNet is not useful to support the more subtle differentiations within a disorder, it needs to be noted that the validity of these subtypes is increasingly criticized [62]. In particular, the validity of the DSM-based subtype model is compromised by minimal empirical support for the distinction between ADHD and ADD on the basis of studies on academic as well as cognitive functioning, treatment responses, and the considerable longitudinal instability of the subtypes [62]. It can therefore not be ruled out that the inability of the applied algorithm to detect differences between the included ADHD subtypes may simply reflect a lack of validity in the clinical distinction between different ADHD subtypes. This supports previous critical appraisals of the validity of distinguishing ADD and ADHD, which overall only seem to find limited evidence for this distinction on the neurophysiological/neuroanatomical level [62,63]. Additionally, it is possible that other deep learning models may perform differently and allows for a better classification of ADHD subgroup. This should be subject to future studies. Similarly, more effort is needed to delineate the boundary conditions under which the applied model is able to perform a reliable classification of individual subjects. Currently, a reliable classification is only possible for some patients/participants and it is unknown what aspects in the EEG data determines whether it is possible or not to perform a reliable classification at the single-subject level.

## 4. Conclusions

This is the first study showing that deep learning methods applied to EEG data are able to dissociate between patients with ADHD and healthy controls. We showed that rather early attentional selection processes associated with superior parietal cortical structures contribute most to classification accuracy. In a subset of patients/controls, the classification accuracy was high (i.e., up to 86%). However, the deep learning approach was not able to distinguish between patients with ADD and ADHD. This may reflect known problems related to the clinical validity of these ADHD subtypes. The results show that deep learning methods based on EEG data represent a promising method for both basic research and clinical applications, but more work needs to be done to increase the reliability of this method for these purposes.

## Figures and Tables

**Table 1:** Details of the EEGNet architecture.

Layer	Layer Type	Filters	Size	Parameters	Output Dimension	Activation	Mode
1	Input				(C, T)		
	Reshape				(1, C, T)		
	Conv2D	F	(1,64)	64*F	(F, C, T)	Linear	same
	BatchNorm			2*F	(F, 1, T)		
	DepthwiseConv2D	F	(C,1)	C*F	(F, 1, T)	Linear	valid
	BatchNorm				(F, 1, T)		
	Activation				(F, 1, T)	ELU	
	SpatialDropout2D				(F, 1, T)		
2	SeparableConv2D	F	(1,8)	$8*F+F^{2}$	(F, 1, T)	Linear	same
	BatchNorm			2*F	(F, 1, T)		
	Activation				(F, 1, T)	ELU	
	AveragePool2D		(1,4)		(F, 1, T//4)		
	SpatialDropout2D				(F, 1, T//4)		
3	SeparableConv2D	2*F	(1,8)	$2*F*8+{\left(2*F\right)}^{2}$	(2*F, 1, T//4)	Linear	same
	BatchNorm			2*F	(2*F, 1, T//4)		
	Activation				(2*F, 1, T//4)	ELU	
	AveragePool2D		(1,4)		(2*F, 1, T//16)		
	SpatialDropout2D				(2*F, 1, T//16)		
4	Flatten				(2*F, 1, T//16)		
	Dense				N	Softmax	

C = number of channels, T = number of time points, F = number of filters and N = number of classes.
